# Evaluation of [^68^Ga]Ga-PSMA PET/CT images acquired with a reduced scan time duration in prostate cancer patients using the digital biograph vision

**DOI:** 10.1186/s13550-021-00765-y

**Published:** 2021-02-28

**Authors:** Manuel Weber, Walter Jentzen, Regina Hofferber, Ken Herrmann, Wolfgang Peter Fendler, Maurizio Conti, Axel Wetter, David Kersting, Christoph Rischpler, Pedro Fragoso Costa

**Affiliations:** 1 Department of Nuclear Medicine, University of Duisburg-Essen and German Cancer Consortium (DKTK)-University Hospital Essen, Hufelandstrasse 55, 45122 Essen, Germany; 2 Siemens Medical Solutions USA, INC, Knoxville, TN USA; 3 Institute of Diagnostic and Interventional Radiology and Neuroradiology, University of Duisburg-Essen, Essen, Germany

**Keywords:** PET/CT, PSMA, Image quality, Silicon photomultiplier

## Abstract

**Aim:**

[^68^Ga]Ga-PSMA-11 PET/CT allows for a superior detection of prostate cancer tissue, especially in the context of a low tumor burden. Digital PET/CT bears the potential of reducing scan time duration/administered tracer activity due to, for instance, its higher sensitivity and improved time coincidence resolution. It might thereby expand [^68^Ga]Ga-PSMA-11 PET/CT that is currently limited by ^68^Ge/^68^Ga-generator yield. Our aim was to clinically evaluate the influence of a reduced scan time duration in combination with different image reconstruction algorithms on the diagnostic performance.

**Methods:**

Twenty prostate cancer patients (11 for biochemical recurrence, 5 for initial staging, 4 for metastatic disease) sequentially underwent [^68^Ga]Ga-PSMA-11 PET/CT on a digital Siemens Biograph Vision. PET data were collected in continuous-bed-motion mode with a mean scan time duration of 16.7 min (reference acquisition protocol) and 4.6 min (reduced acquisition protocol). Four iterative reconstruction algorithms were applied using a time-of-flight (TOF) approach alone or combined with point-spread-function (PSF) correction, each with 2 or 4 iterations. To evaluate the diagnostic performance, the following metrics were chosen: (a) per-region detectability, (b) the tumor maximum and peak standardized uptake values (SUVmax and SUVpeak), and (c) image noise using the liver’s activity distribution.

**Results:**

Overall, 98% of regions (91% of affected regions) were correctly classified in the reduced acquisition protocol independent of the image reconstruction algorithm. Two nodal lesions (each ≤ 4 mm) were not identified (leading to downstaging in 1/20 cases). Mean absolute percentage deviation of SUVmax (SUVpeak) was approximately 9% (6%) for each reconstruction algorithm. The mean image noise increased from 13 to 21% (4 iterations) and from 10 to 15% (2 iterations) for PSF + TOF and TOF images.

**Conclusions:**

High agreement at 3.5-fold reduction of scan time in terms of per-region detection (98% of regions) and image quantification (mean deviation ≤ 10%) was demonstrated; however, small lesions can be missed in about 10% of patients leading to downstaging (T1N0M0 instead of T1N1M0) in 5% of patients. Our results suggest that a reduction of scan time duration or administered [^68^Ga]Ga-PSMA-11 activities can be considered in metastatic patients, where missing small lesions would not impact patient management. Limitations include the small and heterogeneous sample size and the lack of follow-up.

## Introduction

The high sensitivity and specificity of ^68^Ga -labeled prostate-specific membrane antigen-ligand positron-emission tomography ([^68^Ga]Ga-PSMA-11 PET) for prostate cancer lesions has led to an increasing use over the past years [1]. Advantages over other modalities, such as computed tomography (CT) and magnetic resonance imaging as well as bone scan scintigraphy with regards to lesion detection, are particularly marked in patients with low tumor burden, influencing management in a substantial fraction of patients [2, 3].

To ensure optimal image quality the joint EANM/SNMMI procedure guidelines for ^68^Ga-PSMA PET recommend intravenous administration of 1.8–2.2 MBq [^68^Ga]Ga-PSMA-11 per kilogram body weight and an emission time of 2–4 min per bed position in step-and-shoot mode [4]. However, availability of [^68^Ga]Ga-PSMA-11 PET is limited fundamentally by ^68^Ge/^68^Ga-generator yield and, to a lesser degree, positron-emission tomography (PET) scan duration time [5]. Current strategies to expand PSMA PET operation include distribution of ^18^F-labeled probes with longer half-life.

Another approach will be optimization of acquisition techniques, i.e., reducing the administered activity without notable loss of diagnostic performance. Alternatively, a higher patient throughput could be achieved by reducing scan time duration, which would also decrease the risk for radioactive contamination and patient discomfort due to urinary incontinence [6].

A recent study has shown that the administration of a reduced [^68^Ga]Ga-PSMA-11 activity was not feasible without sacrificing tumor detectability and image quality on a “conventional” Siemens Biograph mCT PET/CT system [5]. These limitations might potentially be overcome with the advent of a new generation of “digital” PET/CT systems using digital detector technology (of note, the frequently used term “digital” PET is in a way misleading and can be more aptly replaced by silicon photomultiplier-based PET; however, we adopt the term used in current literature). For example, the digital Biograph Vision PET/CT system allows for a higher detector sensitivity, a higher spatial resolution, and an improved coincidence timing resolution compared with its precedent model, the photomultiplier tube-based Biograph mCT [7, 8]. Phantom and patient studies using ^18^F-labelled glucose have recently confirmed the superior imaging properties of the new system [8]. This might allow for a better detectability of lesions with faint tracer accumulation.

A prior, still unpublished, phantom optimization study (simulating conditions observed for [^68^Ga]Ga-PSMA-11 patients) by our group demonstrated that a three and a half-fold reduction of emission time per bed position did not result in any notable loss of lesion detectability and image quantification when using appropriate image reconstruction algorithms and reconstruction parameters [9]. These results can be projected to the use of low activity protocols, as a reduction of emission time roughly corresponds to a reduction of the administered activity by the same factor [5, 10].

Therefore, the aim of this study was to clinically evaluate the feasibility of a three and a half-fold reduced scan time duration on the digital Biograph Vision with regard to detectability, quantification precision, and image quality. In addition, the impact of different image reconstruction algorithms was evaluated.

## Methods and materials

### Patient population and preparation

Twenty randomly selected patients with prostate cancer undergoing [^68^Ga]Ga-PSMA-11 PET examination (on clinical indication) were included. Mean patient age (range) was 68 (53–78) years, mean (range) prostate-specific antigen (PSA) levels were 26.1 (0.4–258) ng/mL. Additional file 1: Table S1 provides an in-depth overview of the patient characteristics. For [^68^Ga]Ga-PSMA-11 PET performance, a mean ± standard deviation (SD) activity of 124 ± 23 MBq [^68^Ga]Ga-PSMA-11 was injected intravenously. PET/CT data were acquired after a mean ± SD time interval of 58 ± 12 min.

### Image acquisition

All patients were scanned using a digital Biograph Vision PET/CT system (Siemens Healthcare; Erlangen, Germany), which was recently characterized using ^18^F [8]. The [^68^Ga]Ga-PSMA-11 PET examinations included whole-body PET/CT scans from pelvic to the skull base. PET/CT started with a whole-body spiral CT in full-dose technique using automatic tube current and tube voltage adjustments (Care Dose 4D, quality reference 160 mAs; CARE kV, quality reference 120 kV). CT data were used for attenuation correction and anatomical localization. In the absence of contraindications iodinated contrast medium was administered intravenously. Subsequently, two PET scans—a reference acquisition and a reduced acquisition protocol—were applied in continuous-bed-mode. The reference (or clinical standard) scan was acquired first and lasted on average 16.7 min (standard deviation ± 0.6 min). After its completion, the reduced scan was acquired including the same region and lasted on average 4.6 min (standard deviation ± 0.2 min). The mean ± SD time interval between tracer injection and the first and second PET scan time point was 58 ± 12 min 74 ± 12 min, respectively. The three and a half-fold reduction of the scan time duration was based on a still unpublished optimization study performed on the same PET/CT system using an abdominal phantom simulating the prostate region under conditions observed in prostate cancer imaging [9]. The optimized step-and-shoot emission time in this phantom study was 60 s/bed (or 2.19 mm/s in continuous-bed-motion table speed) in association with appropriate image reconstruction algorithms (see below). The conversion from step-and-shoot emission time per bed (*t*_bed_) to continuous-bed-motion table speed (*v*_table_) was based on the manufacturer-recommended equivalence settings using an axial field of view (FOV) of 263 mm (or *v*_table_ = 0.5 FOV / *t*_bed_) [11].

More specifically, our clinical standard protocol comprised two regions, a prostate and a non-prostate region. For the reference acquisition protocol, the continuous-bed-motion table speed (equivalent step-and-shoot emission time per bed position) was 0.6 mm/s (219 s/bed) within the prostate region (scan length of about 30 cm) and 1.2 mm/s (110 s/bed) from the lower abdomen to the skull base (scan length of about 60 cm). The acquisition time of the non-prostate region was slightly shorter than the EANM procedure guidelines recommend, based on the superior imaging properties of the Vision Biograph [7, 8].

For the reduced acquisition protocol, the table speeds for the respective regions were linearly scaled using the ratio of the optimized to standard step-and-shoot emission time for the prostate region (219 s/bed divided by 60 s/bed), that is, the continuous-bed-motion table speed was 2.2 mm/s (60 s/bed) for the prostate region and 4.4 mm/s (30 s/bed) for the non-prostate region, respectively.

### Image reconstruction

The CT images were reconstructed iteratively with a convolution kernel I30f (SAFIRE level of 3). The reconstructed CT slice thickness and the transversal voxel size was 3 mm and 1.5 × 1.5 mm^2^, respectively. In reference to the phantom optimization study, PET images were reconstructed using the three-dimensional ordinary Poisson ordered-subset expectation maximization (OSEM) algorithm with time-of-flight (TOF) approach alone or combined with point-spread-function (PSF) correction [9]. All reconstructions included scatter and CT-based attenuation correction, decay correction, normalization, and correction for random coincidence. Scatter was corrected using the extended single-scatter simulation algorithm, which distinguished the scattered annihilation radiation according to its TOF [8]. In addition, a prompt gamma coincidence correction method is by default implemented in the PET reconstruction algorithm for radionuclides emitting prompt gammas such as ^68^Ga (branching ratio of 89% and prompt gamma fraction of 1.2%) [12]. For both acquisition protocols, four image sets were reconstructed: TOF and TOF + PSF, each with 2 iterations (5 subsets) or 4 iterations (5 subsets). The reconstructed images had a voxel size of 3.3 × 3.3 × 3.0 mm^3^ and were smoothed with an isotropic Gaussian post‐reconstruction filter of 4 mm [9]. The measured reconstructed PET spatial resolution (expressed as the full-width-at-half maximum) was 6.2 mm and 5.6 mm for TOF- and TOF + PSF-reconstructed images, respectively [9]. The resulting 4 images (reconstructed for each patient and each acquisition protocol) are referred to OSEM-TOF(2i), OSEM-TOF(4i), OSEM-TOF + PSF (2i), and OSEM-TOF + PSF(4i).

### Image analyses

PET data sets were pseudonymized and evaluated in random order by a blinded reader (with no image acquisition and reconstruction information as well as no clinical information). Focal PSMA-uptake higher than the surrounding background was classified as neoplastic if not associated with physiological organ uptake [13]. Pathological findings were then divided into four separate body regions (local tumor, regional lymph node metastases, and soft tissue metastases including extrapelvic lymph nodes and bone metastases) [14]. Maximum and peak standardized uptake values (SUVmax and SUVpeak) were measured for the tumor with the most intense tracer uptake in each body region. Additionally, in each patient, one lesion with faint tracer uptake (if available) was measured. Reading results were then compiled by a member not involved in the reading process. A joint consensus session by two physicians was performed for discordant reports between series of the same patient. Due to the published high inter-observer agreement for [^68^Ga]Ga-PSMA-11 PET reporting by multiple blinded readers was not deemed necessary [15]. In addition, a spherical volume of interest with a diameter of 30 mm was drawn within the inferior right lob of the liver; the SD of the liver’s tissue activity distribution and its mean was ascertained for image noise evaluation [16, 17].

### Metrics for diagnostic performance

Three metrics were used to evaluate the diagnostic performance. Primary endpoint was the accuracy of the per-region detectability in the images acquired with the reduced protocol. To this end, images reconstructed with the same algorithm, but acquired with standard emission time duration, were set as reference image. The percentage fraction of correctly classified tumor regions in the images using the reduced acquisition was calculated and changes of miTNM stage were assessed. As secondary endpoint, the precision in image quantification was evaluated using SUVmax and SUVpeak. The ratio between SUVmax (SUVpeak) of PSMA-positive tumors in the reduced and reference acquisition protocol series was calculated among the respective image reconstruction algorithms. The resulting SUV ratios were further categorized in terms of SUVmax showing tumors with faint (SUVmax ≤ 5), moderate (5 < SUVmax < 30), and high uptake (SUVmax ≥ 30). The SUVmax (SUVpeak) among reference and reduced protocols were correlated by using Pearson product-moment correlation. For the same pairs, Bland–Altman analysis was used to determine the mean differences and 95% limits of agreement between the differences. Finally, the third metric evaluates the image noise and was the percentage ratio of the SD of the tissue activity distribution within the selected liver VOI to its mean value.

## Results

### Detectability

As assessed by the reference protocol 14/20 patients (70%) were PSMA PET-positive, 8/20 (40%) patients had local tumor, 3/20 (15%) pelvic lymph node metastases, 5/20 (25%) extrapelvic lymph node metastases, 7/20 (35%) bone metastases. None of the patients had visceral metastases. Additionally, focal [^68^Ga]Ga-PSMA uptake in a celiac ganglion, representing a common pitfall was visible across all acquisition and reconstruction protocols [18].

Using series acquired with the reference protocol, 78/80 regions (98%) and 21/23 (91%) regions with at least one tumour lesion were correctly classified in the reduced protocol (Additional file 1: Tables S2 and S3). Additional file 1: Table S4 gives an extensive overview of patient characteristics including miTNM stage [19] for both protocols. No differences regarding the region classification were observed between the different reconstruction algorithms.

**Fig. 1:**
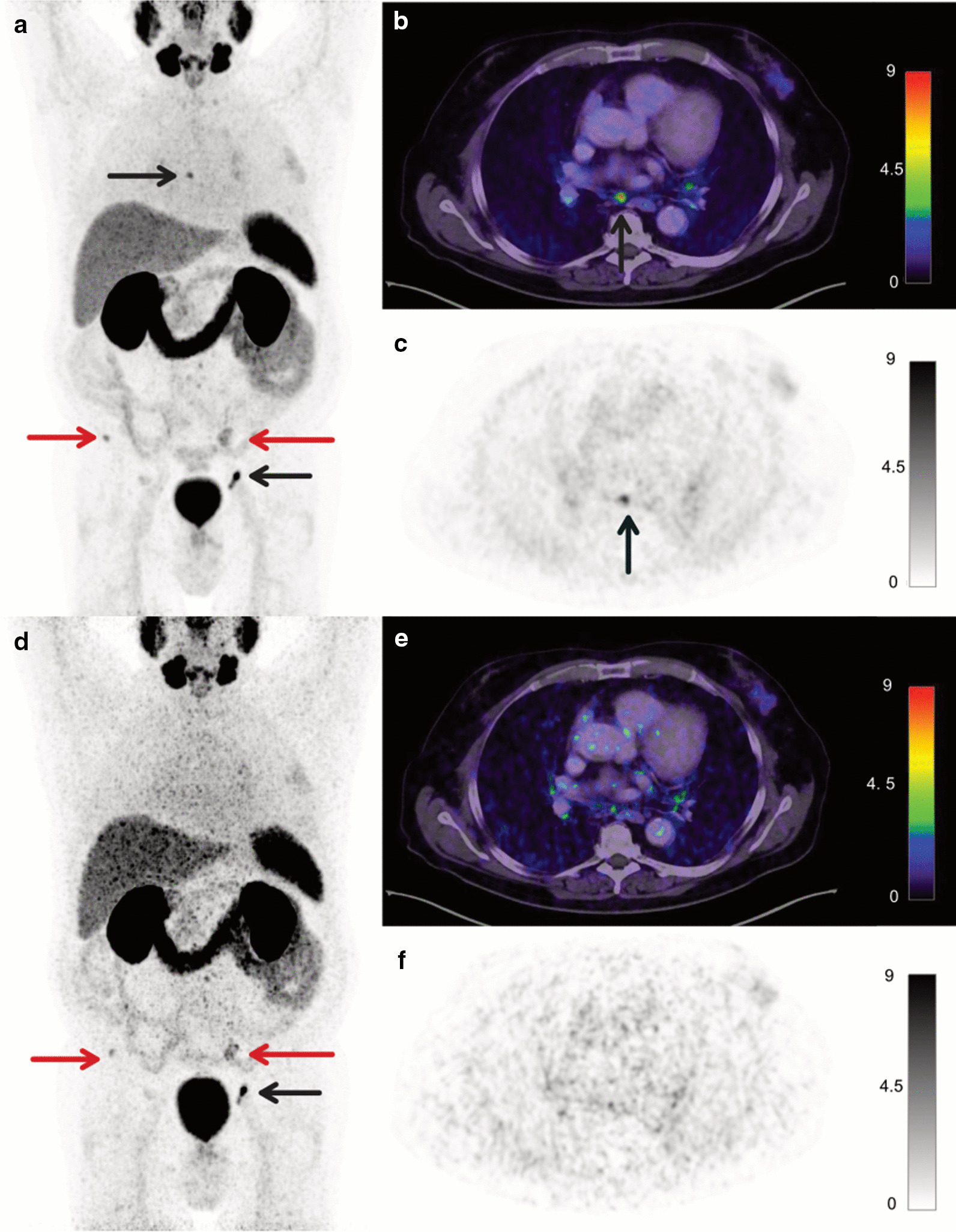
A 65-years old patient (Pat. ID #7) with second biochemical recurrence after primary prostatectomy and salvage external beam radiation therapy. PSA was 0.6 ng/ml at the time of imaging. **a**–**c** show images acquired with the reference acquisition protocol; **d**–**f** show images acquired with the reduced acquisition protocol, all reconstructed with OSEM-PSF + TOF(4i). Pathological tracer uptake in a mediastinal lymph node visible on the images acquired with reference protocol (**c**, black arrow) was not reproducible with three and a half-fold reduction in scan time duration (**f**). Maximum intensity projection (**a**, **d**) and axial [^68^Ga]Ga-PSMA-11 PET/CT slices (**b**, **c**, **e**, **f**) show pelvic and extrapelvic lymph node metastases (black arrows) and bone metastases (red arrows)

**Fig. 2:**
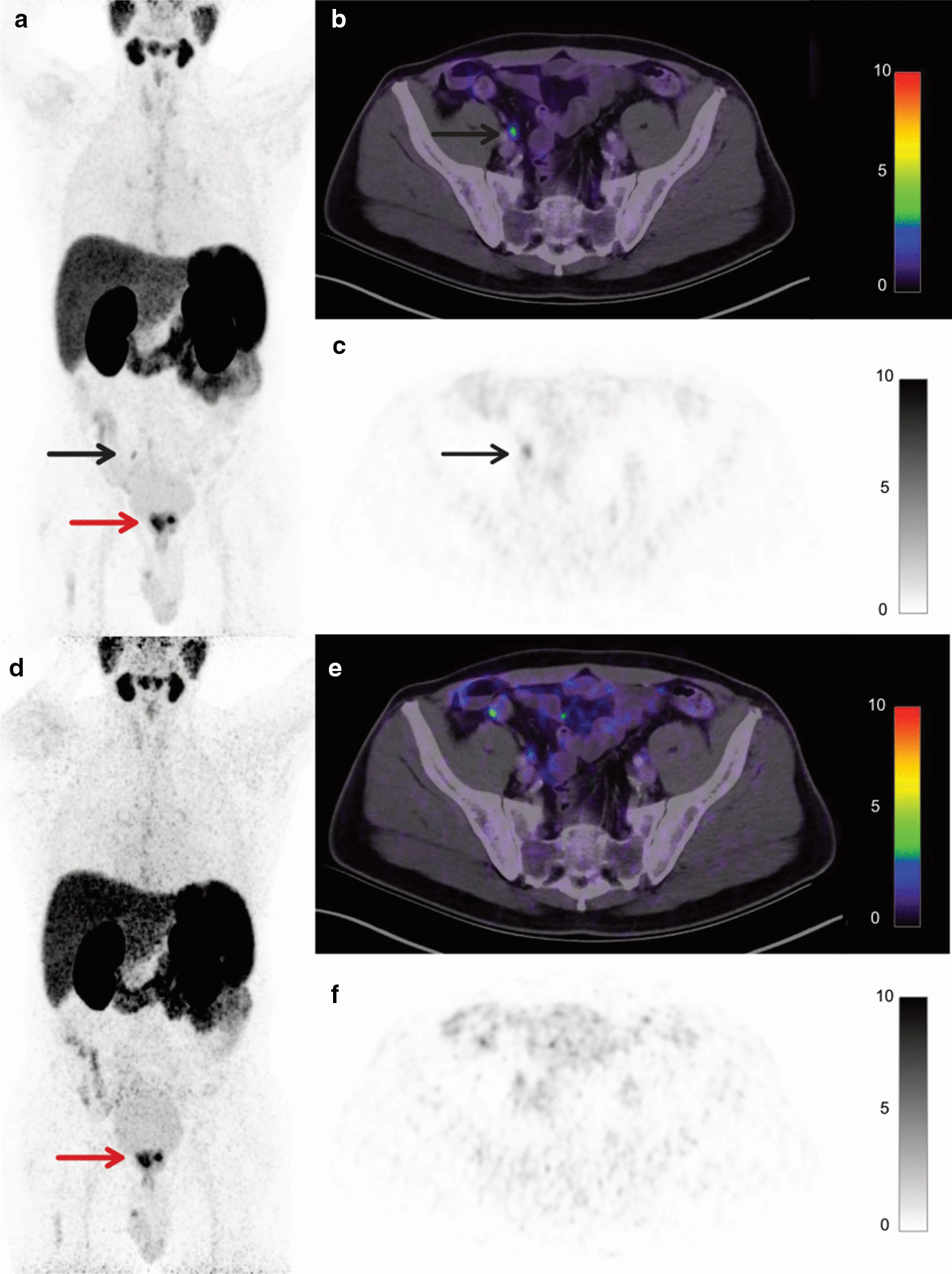
A 61-year-old patient (Pat ID #13) with biopsy-proven prostate cancer undergoing [^68^Ga]Ga-PSMA-11 PET/CT for initial tumor staging before treatment. Gleason Score was 9, PSA was 18.3 ng/ml at the time of imaging. **a**–**c** show images acquired with the reference acquisition protocol; **d**–**f** show images acquired with the reduced acquisition protocol all reconstructed with OSEM-PSF + TOF(4i). Maximum intensity projection (**a**, **d**) and axial [^68^Ga]Ga-PSMA-11 PET/CT slices (**b**, **c**, **e**, **f**) show local tumor (red arrows). One right pelvic lymph node metastasis (black arrow) could not be unequivocally detected with a three and a half-fold scan time reduction (**c**, **f**). Until now the patient has not undergone surgery, a follow-up scan performed more than 6 months later confirmed the prostatic and lymph node lesions

In two patients, one small nodal lesion (each ≤ 4 mm short-axis diameter derived from CT images) was missed, each impacting miTNM stage (T1N0M0 instead of T1N1M0; T0N1M1b instead of T0N1M1aM1b). SUVmax (SUVpeak) of the missed mediastinal lymph node (Fig. 1) was 5.7 (3.1), SUVmax (SUVpeak) of the missed pelvic lymph node (Fig. 2) was 3.8 (2.2). However, just one of these missed lesions led to clinically relevant downstaging.

Figures 1 and 2 show [^68^Ga]Ga-PSMA-11 PET/CT images of the patients, in whom the lesions were missed across all reconstruction algorithms.

### Image quantification

**Fig. 3:**
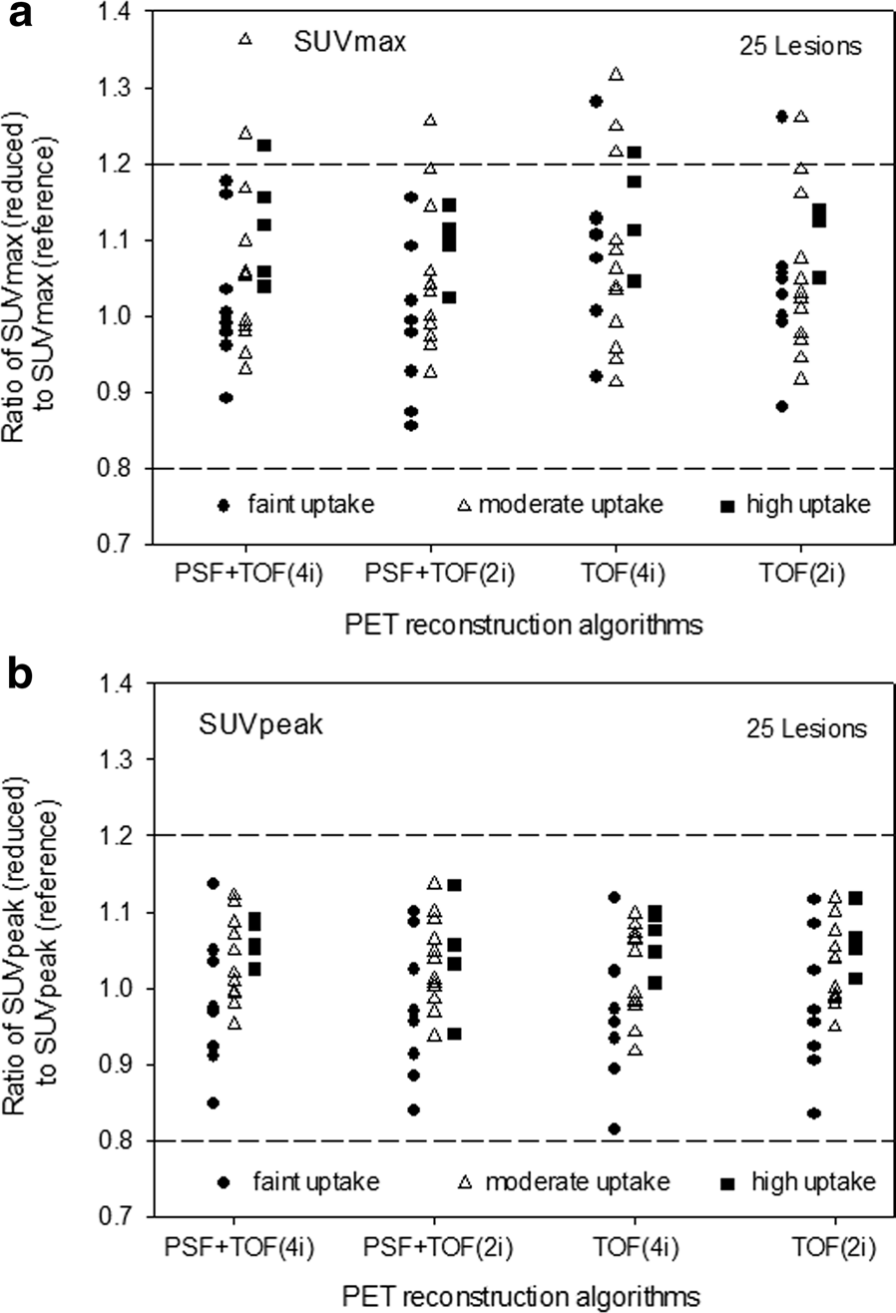
Dot plots showing the ratio of SUVmax (**a**) and SUVpeak (**b**) between images acquired with reference and reduced scan duration, separately for lesions with faint (filled dots), moderate (white triangles) and intense tracer uptake (filled squares). Margins (± 20) are shown in dashed lines

**Fig. 4:**
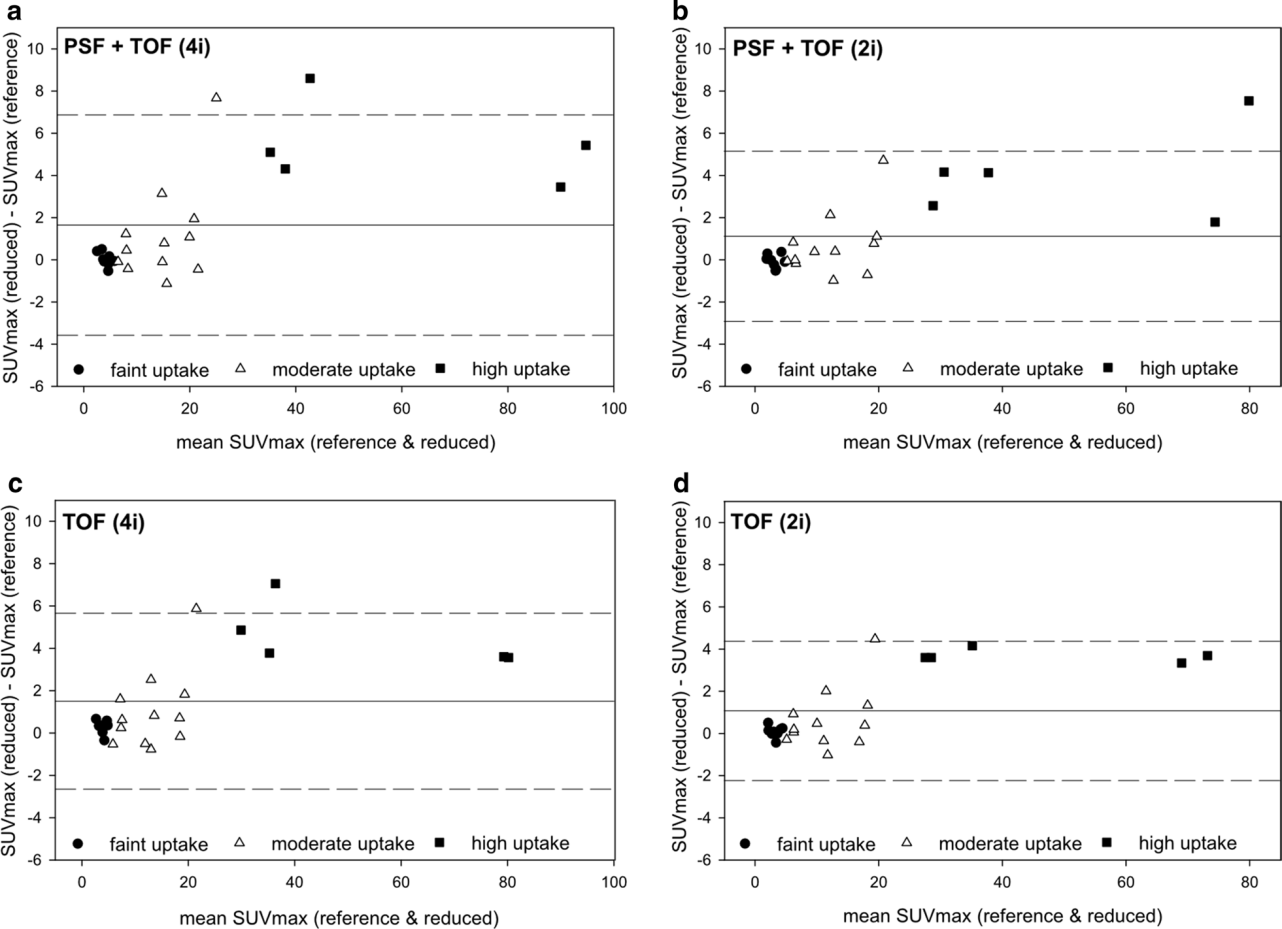
Bland–Altman plots showing the agreement of SUVmax between images acquired with reduced vs. standard emission time reconstructed with PSF + TOF (4i, panel **a**) PSF + TOF (2i, panel **b**), TOF (4i, panel **c**), and TOF (2i, panel **d**). A systematic overestimation of high-uptake lesions was observed

**Fig. 5:**
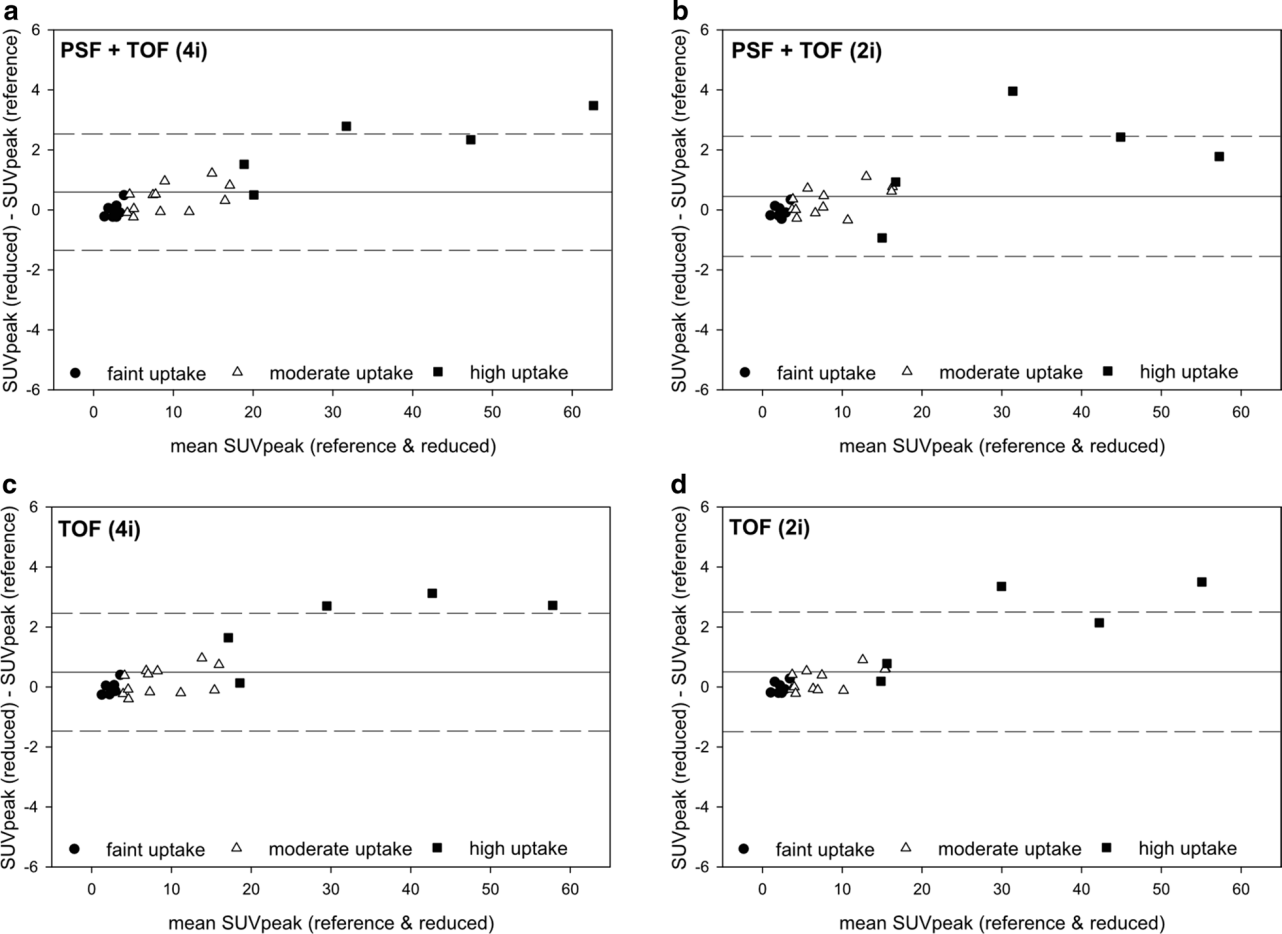
Bland–Altman plots showing the agreement of SUVpeak between images acquired with reduced vs. standard emission time reconstructed with PSF + TOF (4i, panel **a**) PSF+TOF (2i, panel **b**), TOF (4i, panel **c**), and TOF (2i, panel **d**). A systematic overestimation of high-uptake lesions was observed

In Fig. 3, dot plots of the ratio of SUVmax und SUVpeak for all 25 lesions are provided separately for lesions with faint, moderate and high uptake. SUVs between images acquired with reduced *vs*. standard protocol images reconstructed with the same algorithms differed by less than 20%, which we defined as an acceptable error margin. The mean absolute percentage deviation (including all 25 lesions) for SUVmax (SUVpeak) for the different image algorithms were 9.4% (6.1%), 8.1% (6.4%), 11.2% (6.2%), 8.3% (5.9%) for OSEM-TOF + PSF (4i), OSEM-TOF + PSF (2i), OSEM-TOF (4i), OSEM-TOF (2i), respectively. No notable differences (≤ 20%) were observed when comparing lesions with different uptake intensities (faint, moderate, and high). Pearson’s correlation coefficient between SUVmax of standard vs. reduced acquisition time was 0.996 (PSF + TOF 4i), 0.998 (PSF + TOF 2i), 0.997 (TOF 4i), 0.998 (TOF 2i), respectively. For SUVpeak Pearson’s correlation coefficient was 1.000 (PSF + TOF 4i), 0.999 (PSF + TOF 2i), 0.999 (TOF 4i), 0.999 (TOF 2i), respectively. All Pearson coefficients were significantly correlated (*p* < 0.01). The Bland–Altman plot shows systematic overestimation of SUVmax and SUVpeak in the images acquired with reduced acquisition time (Figs. 4 and 5). This overestimation was more pronounced when using SUVmax. Outliers and scatter levels appear to be more pronounced in images reconstructed with 4 as opposed to 2 iterations.

### Image noise

**Fig. 6:**
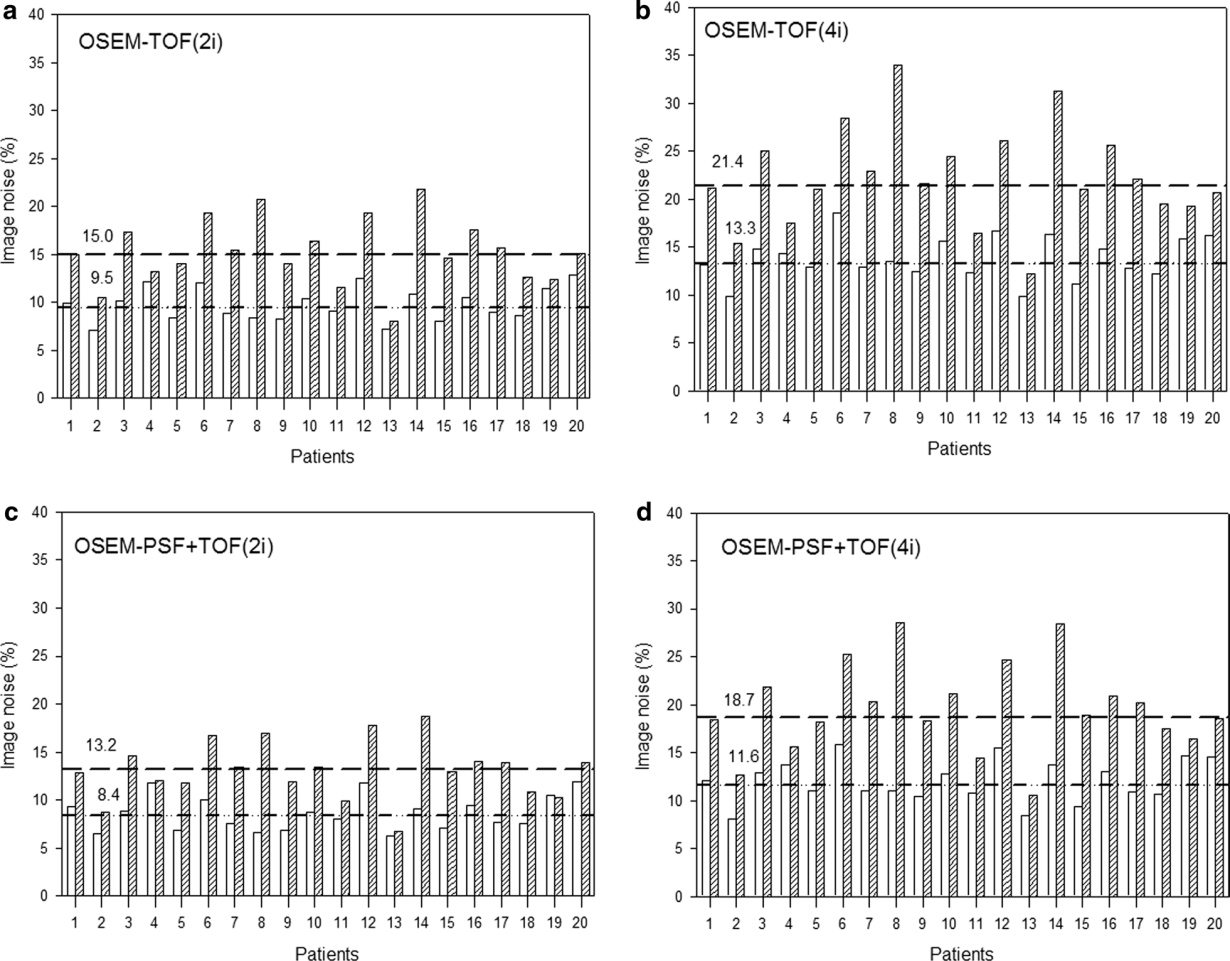
Bar chart showing the image noise derived from the liver’s activity distribution for images acquired with the reference (white bars) and reduced protocol (hatched bars) and reconstructed with different reconstruction algorithms (**a**–**d**). Medians are illustrated using dashed lines and the respective numbers are close to the lines

The mean image noise was higher for images acquired with the reduced protocol than for images acquired with the reference protocol (Fig. 6) and these differences were most pronounced in the images reconstructed with 4 iterations. The mean image noise increased from 12 to 20% for OSEM-TOF + PSF (4i), 9% to 13% for OSEM-TOF + PSF (2i), 14% to 22% for OSEM-TOF (4i) and 10% to 15% for OSEM-TOF (2i).

## Discussion

This study indicates that a reduction of the scan time duration or administered [^68^Ga]Ga-PSMA-11 activity produces results comparable to the reference acquisition protocol on a digital Biograph Vision PET/CT system both for detectability (98% of regions correctly identified) and image quantification (mean absolute deviation ≤ 10%) for all reconstruction algorithms but OSEM-TOF (4i).

In our cohort of 20 prostate cancer patients across a variety of miTNM stages undergoing [^68^Ga]Ga-PSMA-11 PET, only two small nodal lesions (short-axis diameter of ≤ 4 mm) were missed (Figs. 1 und 2) leading to miTNM downstaging in one case. The first lesion was located close to the right common iliac artery, the second in the mediastinum. In one of these cases (lesion 1), this downstaging would have possibly impacted patient management negatively, in the other (lesion 2) the reduced emission time would have been unlikely to cause major changes in management as the patient also had bone metastases.

However, low sensitivities of PSMA PET performed with “conventional” PET/CT systems have previously been reported for the detection of small lesions (< 5 mm) due to partial volume effects [20]. Both of the missed lesions in our cohort showed moderate to faint tracer uptake (SUVmax 5.7 and SUVmax 3.8), which is also known to negatively affect detectability [21]. Additionally, high background due to unspecific small intestinal or mediastinal uptake considerably hampered lesion detection (Figs. 1 and 2). A further possible explanation would be motion artifacts, among others caused by gastrointestinal peristaltic and arterial pulsation. Administered activities for these patients were above average (129 MBq, 135 MBq) and uptake time within one standard deviation of the mean (48 and 55 min); therefore, both factors are unlikely to be causal.

The main drawback of our reduced acquisition protocol is that small nodal lesions could be missed leading to false-negative [^68^Ga]Ga-PSMA-11 PET reports, especially in the non-prostate lesion, where the acquisition time is particularly short. This could be addressed by optimizing patient selection, and performing reduced activity protocols in patients, in whom missing small nodal lesions would not impact management. One example could be the imaging of patients with known remote metastases (although the appearance of small new lesions could be missed) and/or those before PSMA-directed radioligand therapy. On the other hand, patients with suspected low tumor burden and/or patients at initial diagnosis would not be ideal candidates.

Recently, two studies have been published that tried to optimize the administered activity and to reduce the emission time duration. First, our findings differ from a previous trial by Rauscher et al. [5], who showed unsatisfactory results for list-mode reconstructed images simulating the administration of one-third and two-third of the standard activity. In contrast to their methodology, whole-body PET list-mode reconstruction was not applicable in our study due to the use of continuous-bed-motion mode, which should preferably be used if available [22]. Additionally, patients enrolled in the trial by Rauscher et al. [5] underwent [^68^Ga]Ga-PSMA-11 PET on a Siemens Biograph mCT, so the discrepancy in findings might potentially be explained by the different imaging characteristics when compared to its successor, the Siemens Biograph Vision. Second, van Sluis et al. [10] showed an improvement in visually assessed image quality, tumor lesion demarcation, and overall image quality in oncological patients undergoing 2-[^18^F]FDG PET/CT [7]. In agreement with our short acquisition protocol the same group [10] also found that a threefold reduction of administered activity in oncological patients was feasible, with TNM down-staging only occurring in 1/30 patient cases.

No differences with regards to the detectability were observed for the different reconstruction algorithms. Of note, additional PSF reconstruction did not provide additional value in terms of detectability. This can be largely explained by the implementation of a 4-mm Gaussian filter, producing similar PET reconstructed spatial resolutions for TOF- and TOF + PSF-reconstructed images (6.2 mm vs. 5.6 mm) [9]. In addition, under reduced statistical conditions, PET images will inevitably display higher noise [23]. To compensate this loss in image quality, a careful adaptation of iteration number could be considered without compromising lesion detectability by insufficient iterative convergence. In fact, our data suggests that with TOF and TOF + PSF modelling, image noise in the liver can be reduced by applying 2 iterations instead of 4 (Fig. 6). This results confirm previous investigations, underlining the fast convergence capability of TOF [24]. Additionally, recent publications suggest that the implementation of machine learning approaches might enable the image reconstruction of standard activity images even when very low activities are used [25].

There are several limitations in this study. A limitation of our study is the relatively small and heterogeneous sample size, encompassing patients with a wide variety of miTNM stages. Additionally, the reduced acquisition protocol was applied after the reference acquisition protocol and uptake intervals were quite heterogeneous. On the one hand, by doing so the radionuclide decay occurring in the meantime as well as the better alignment with the CT scan favor the standard protocol. On the other hand, metabolic activity changes can occur between both scan acquisitions. As prior studies have observed an increase in tumoral PSMA uptake between images acquired 3 h after tracer administration versus 1 h after tracer administration, this might have contributed to differences in image quantification (i.e., the observed overestimation for a few lesions) [26]. This leads to the alternative hypothesis that lesions inapparent on the images acquired (later) with the reduced acquisition protocol, might have been non-neoplastic lesions with decreasing PSMA-uptake over time. Therefore, the reason for missing them might rather be the later uptake interval than the reduced acquisition time.

Furthermore, detectability was performed on a per-region level instead of a per-lesion level. Since the per-region analysis does not account for the identification of additional lesions in the standard acquisition protocol in a region that is already rated positive in both acquisition protocols, the per-lesion detectability is potentially lower. As the scan time duration of the non-prostate region in the clinical protocol was slightly below the 2–4 min recommended by the EANM guideline, patients might also have potentially been understaged by the clinical protocol. A further limitation of this study is that lesion validation was not performed. However, the current literature suggests a high positive predictive value of [^68^Ga]Ga-PSMA-11 PET making this a minor issue [3].

## Conclusion

This study shows that the advent of a new generation of digital PET/CT systems bears the potential of reducing emission time (or administered activity) while maintaining an acceptable level of diagnostic performance, As small lesions can be missed, a potential application of a reduced activity/reduced emission time protocol is the imaging of metastatic patients in whom missing small nodal lesions would not impact patient management. As ^68^Ge/^68^Ga-generator yield is currently the main limiting factor in most imaging sites, an optimized protocol may subsequently considerably improve [^68^Ga]Ga-PSMA-11 PET availability.

## Supplementary Information

**Additional file 1**. Patient characteristics, details on lesion detection per region, and resulting stage migration.

## Data Availability

The datasets generated and/or analyzed during the current study are not publicly available due to privacy legislation but are available from the corresponding author on reasonable request.
