# Supplementary material for: Evaluation of [68Ga]Ga-PSMA PET/CT images acquired with a reduced scan time duration in prostate cancer patients using the digital biograph vision
Source: EJNMMI Res. 2021 Feb 28;11:21. doi: 10.1186/s13550-021-00765-y (PMC7914332; doi:10.1186/s13550-021-00765-y)
Supplement: Supplementary file 1 — Additional file 1. Patient characteristics, details on lesion detection per region, and resulting stage migration. [file 13550_2021_765_MOESM1_ESM.docx]

## **Supplemental Table S1**

| **Table S1.**  Patient Characteristics (n=20) | |
| --- | --- |
| Age and PSA | Values for age and PSA |
| Median (range) age (years) | 68 (53–78) |
| Median (range) PSA (ng/mL) | 3.2 (0.4–258) |
|  |  |
| Gleason score | Number of patients |
| 6  7  8  9  NA | 4  5  3  3  5 |
|  |  |
| Initial treatment (number of patients) | Number of patients |
| None  Prostatectomy  Primary EBRT  Salvage EBRT  ADT  Enzalutamide/Abiraterone  Chemotherapy  Others | 5  11  3  5  5  4  3  1 |
| NA, not available; EBRT, external beam radiation therapy | |

## **Supplemental Table S2**

| Overview of the lesion detectability using images reconstructed with OSEM-TOF 4i (served as reference) in comparison with the detectability using images reconstructed with OSEM-TOF 4i (short) and OSEM-TOF 2i (reduced). | | | |
| --- | --- | --- | --- |
| Pat. ID. | Lesion detection  OSEM-TOF(4i) (reference) | Lesion detection  OSEM-TOF(4i) ( reduced) | Lesion detection  OSEM-TOF(2i) (reduced) |
| 1 | 1/0/1/1 | 1/0/1/1 | 1/0/1/1 |
| 2 | 0/0/1/0 | 0/0/1/0 | 0/0/1/0 |
| 3 | 0/0/0/0 | 0/0/0/0 | 0/0/0/0 |
| 4 | 0/0/0/0 | 0/0/0/0 | 0/0/0/0 |
| 5 | 1/0/0/0 | 1/0/0/0 | 1/0/0/0 |
| 6 | 0/0/1/0 | 0/0/1/0 | 0/0/1/0 |
| 7 | 0/1/1/1 | 0/1/**0**/1 | 0/1/**0**/1 |
| 8 | 1/0/0/0 | 1/0/0/0 | 1/0/0/0 |
| 9 | 0/0/0/1 | 0/0/0/1 | 0/0/0/1 |
| 10 | 0/0/0/1 | 0/0/0/1 | 0/0/0/1 |
| 11 | 0/0/0/0 | 0/0/0/0 | 0/0/0/0 |
| 12 | 0/0/0/0 | 0/0/0/0 | 0/0/0/0 |
| 13 | 1/1/0/0 | 1/**0**/0/0 | 1/**0**/0/0 |
| 14 | 1/1/1/1 | 1/1/1/1 | 1/1/1/1 |
| 15 | 1/0/0/0 | 1/0/0/0 | 1/0/0/0 |
| 16 | 0/0/0/1 | 0/0/0/1 | 0/0/0/1 |
| 17 | 1/0/0/0 | 1/0/0/0 | 1/0/0/0 |
| 18 | 1/0/0/1 | 1/0/0/1 | 1/0/0/1 |
| 19 | 0/0/0/0 | 0/0/0/0 | 0/0/0/0 |
| 20 | 0/0/0/0 | 0/0/0/0 | 0/0/0/0 |
| *Classified tumor regions are local tumor/regional metastatic lymph nodes/extrapelvic soft-tissue tumors including lymph nodes/bone tumors. Symbol „1“ marks the presence of at least one lesion, symbol „0“ denotes the absence of lesions in this region. Mismatches in comparison with the reference are marked in red. | | | |

## **Supplemental Table S3**

| **Table S3**  Overview of the lesion detectability using images reconstructed with OSEM-TOF+PSF(4i) (served as as reference) in comparison with the detectability using images reconstructed with OSEM-TOF+PSF(4i) (reduced) and OSEM-TOF+PSF(2i) (reduced). | | | |
| --- | --- | --- | --- |
| Pat. ID | Lesion detection OSEM-PSF+TOF(4i)  (reference) | Lesion detection OSEM-PSF+TOF(4i) (reduced) | Lesion detection OSEM-PSF+TOF(2i) (reduced) |
| 1 | 1/0/1/1 | 1/0/1/1 | 1/0/1/1 |
| 2 | 0/0/1/0 | 0/0/1/0 | 0/0/1/0 |
| 3 | 0/0/0/0 | 0/0/0/0 | 0/0/0/0 |
| 4 | 0/0/0/0 | 0/0/0/0 | 0/0/0/0 |
| 5 | 1/0/0/0 | 1/0/0/0 | 1/0/0/0 |
| 6 | 0/0/1/0 | 0/0/1/0 | 0/0/1/0 |
| 7 | 0/1/1/1 | 0/1/**0**/1 | 0/1/**0**/1 |
| 8 | 1/0/0/0 | 1/0/0/0 | 1/0/0/0 |
| 9 | 0/0/0/1 | 0/0/0/1 | 0/0/0/1 |
| 10 | 0/0/0/1 | 0/0/0/1 | 0/0/0/1 |
| 11 | 0/0/0/0 | 0/0/0/0 | 0/0/0/0 |
| 12 | 0/0/0/0 | 0/0/0/0 | 0/0/0/0 |
| 13 | 1/1/0/0 | 1/**0**/0/0 | 1/**0**/0/0 |
| 14 | 1/1/1/1 | 1/1/1/1 | 1/1/1/1 |
| 15 | 1/0/0/0 | 1/0/0/0 | 1/0/0/0 |
| 16 | 0/0/0/1 | 0/0/0/1 | 0/0/0/1 |
| 17 | 1/0/0/0 | 1/0/0/0 | 1/0/0/0 |
| 18 | 1/0/0/1 | 1/0/0/1 | 1/0/0/1 |
| 19 | 0/0/0/0 | 0/0/0/0 | 0/0/0/0 |
| 20 | 0/0/0/0 | 0/0/0/0 | 0/0/0/0 |
| *Classified tumor regions are local tumor/regional metastatic lymph nodes/extrapelvic soft-tissue tumors including lymph nodes/bone tumors. Symbol „1“ marks the presence of at least one lesion, symbol „0“ denotes the absence of lesions in this region. Mismatches in comparison with the reference are marked in red. | | | |

## **Supplemental Table S4**

**Table S4.**
Imaging Characteristics Including miTNM Stage as Assessed by the Reference and Reduced Protocol

| Pat. ID | Activity  (MBq) | | | Uptake  period (min) | Weight (kg) | Reference  protocol | Reduced  protocol |
| --- | --- | --- | --- | --- | --- | --- | --- |
| 1 | | 124 | | 45 | 75 | T1N1M1aM1b | T1N1M1aM1b |
| 2 | | 155 | | 63 | 94 | T0N1M1a | T0N1M1a |
| 3 | | 86 | | 32 | 79 | T1N0 | T1N0 |
| 4 | | 94 | | 57 | 83 | T1N0 | T1N0 |
| 5 | | 134 | | 48 | 94 | T1N0 | T1N0 |
| 6 | | 119 | | 65 | 76 | T0N0M1a | T0N0M1a |
| 7 | | 129 | | 48 | 75 | T0N1M1aM1b | **T0N1M1b** |
| 8 | | 151 | | 61 | 75 | T1N0 | T1N0 |
| 9 | | 135 | | 43 | 97 | T1N0M1b | T1N0M1b |
| 10 | | 124 | | 60 | 110 | T0N0M1bM1c | T0N0M1bM1c |
| 11 | | 152 | | 68 | 72 | T1N0 | T1N0 |
| 12 | | 148 | | 68 | 80 | T1N0 | T1N0 |
| 13 | | 135 | | 55 | 92 | T1N1 | **T1N0** |
| 14 | | 87 | | 46 | 80 | T1N1M1aM1b | T1N1M1aM1b |
| 15 | | 128 | | 72 | 81 | T1N0 | T1N0 |
| 16 | | 88 | | 62 | 95 | T0N0M1b | T0N0M1b |
| 17 | | 93 | | 82 | 72 | T1N0 | T1N0 |
| 18 | | 128 | | 42 | 87 | T1N0M1b | T1N0M1b |
| 19 | | 134 | | 70 | 77 | T0N1 | T0N1 |
| 20 | | 137 | | 71 | 100 | T0N0 | T0N0 |
|  | | | Pat. ID, patient identification; changes in miTNM stage are marked in red. | | | | |
